# Lessons Learned about Neurodegeneration from Microglia and Monocyte Depletion Studies

**DOI:** 10.3389/fnagi.2017.00234

**Published:** 2017-07-28

**Authors:** Harald Lund, Melanie Pieber, Robert A. Harris

**Affiliations:** Department of Clinical Neuroscience, Karolinska Institutet, Centre for Molecular Medicine, Karolinska Hospital at Solna Solna, Sweden

**Keywords:** microglia, monocyte, neurodegeneration, depletion, experimental models in neuroscience

## Abstract

While bone marrow-derived Ly6C^hi^ monocytes can infiltrate the central nervous system (CNS) they are developmentally and functionally distinct from resident microglia. Our understanding of the relative importance of these two populations in the distinct processes of pathogenesis and resolution of inflammation during neurodegenerative disorders was limited by a lack of tools to specifically manipulate each cell type. During recent years, the development of experimental cell-specific depletion models has enabled this issue to be addressed. Herein we compare and contrast the different depletion approaches that have been used, focusing on the respective functionalities of microglia and monocyte-derived macrophages in a range of neurodegenerative disease states, and discuss their prospects for immunotherapy.

## Peripheral and Resident Myeloid Cell Populations

“Myeloid cell” is a collective term that encompasses many innate immune cells including macrophages and monocytes. Resident macrophages are specialized subsets defined by their organ location (e.g., central nervous system (CNS) microglia, liver Kupffer cells), having been differentiated in these specialized microenvironments during development from (in the majority of cases) embryonic precursors. They primarily conduct homeostatic functions in the tissues, but their numbers and properties can change during a local inflammatory process, and they can proliferate within tissues in order to maintain homeostasis. In contrast, circulating monocytes are derived from hematopoietic stem cells in the bone marrow and are generally divided into two subsets in the blood of mice based on their expression of Ly6C. Ly6C^low^ monocytes have endothelium patrolling and scavenging functions whereas Ly6C^hi^ monocytes infiltrate tissue in response to inflammation and differentiate into macrophages. Monocytes are constantly turned over and generally have limited life spans in the circulation.

## Microglia Depletion Models

Depletion of CNS-resident or infiltrating microglia/macrophage cell populations has been used to dissect the role of these cell types during neurodegenerative disorders. The major advances in developing microglia/macrophage depletion tools are summarized below, but for a more detailed review the reader is referred to Waisman et al. (2015), Wieghofer et al. (2015) and Jäkel and Dimou (2017). Depletion of macrophage populations first became a readily available technique through the development of clodronate-encapsulated liposomes in the 1980s (van Rooijen, 1989). Intravenous administration efficiently depleted circulating monocytes (Sunderkötter et al., 2004) and since they did not pass the blood brain barrier this technique could be used to ablate circulating monocytes without affecting CNS-resident macrophage populations (Zattoni et al., 2011). More specific targeting of Ly6C^hi^ monocytes can be accomplished by antibody-mediated depletion (Mack et al., 2001), or by the use of CCR2^−/−^ mice in which Ly6C^hi^ monocytes are unable to leave the bone marrow (Serbina and Pamer, 2006). Intracerebroventricular (i.c.v) delivery of clodronate liposomes has also been used to deplete microglia (Lee et al., 2012; Hanafy, 2013; Asai et al., 2015). In addition, i.c.v injection of mannosylated liposomes is an established technique to specifically target mannose receptor (CD206)-expressing perivascular macrophages without affecting resident microglia numbers in both rats (Polfliet et al., 2001; Newman et al., 2005) and mice (Galea et al., 2005; Hawkes and McLaurin, 2009).

A major breakthrough in the development of efficient microglia depletion tools was the development of mice expressing the herpes-simplex virus encoded suicide-gene thymidine kinase (HSVTK) under the CD11b-promoter (Heppner et al., 2005). Administration of ganciclovir via an osmotic pump-connected i.c.v cannula resulted in up to 95% depletion of microglia (Grathwohl et al., 2009) with the only drawback being that drug administration became toxic after extended delivery, thereby limiting this approach to a period of 4 weeks. In subsequent studies, it was demonstrated that cessation of ganciclovir delivery resulted in complete exchange of the microglial pool by peripheral myeloid cells (Varvel et al., 2012, 2015; Prokop et al., 2015). A major step forward in achieving more specific microglia targeting was the development of CX3CR1^CreER^ mice (Goldmann et al., 2013; Parkhurst et al., 2013). When bred with Rosa26^DTR^ mice and subsequent to peripheral administration of the drug tamoxifen followed by diphtheria toxin, efficient depletion of microglia resulted without affecting bone marrow-derived CX3CR1^+^ cells (Parkhurst et al., 2013; Bruttger et al., 2015). Since other CNS-associated (perivascular, meningeal and choroid plexus) macrophages also express CX3CR1 (Goldmann et al., 2016), these cells are most likely also depleted in CX3CR1^CreER^Rosa26^DTR^ mice. Transcriptional profiling has revealed genes uniquely expressed by microglia such as *Sall1*, and this has been successfully utilized in Sall1^CreER^ mice which target microglia while sparing both peripheral and CNS-associated macrophage populations (Buttgereit et al., 2016). However, Sall1^CreER^ mice have currently not been used to deplete microglia.

Microglia require CSF1R during development, since CSF1R^−/−^ mice completely lack microglia (Ginhoux et al., 2010). Microglia can use both ligands of CSF1R for their survival (CSF1 and IL-34) since mice mutant for either cytokine display reduction but not complete loss of microglia (Ginhoux et al., 2010; Greter et al., 2012; Wang et al., 2012). Elmore et al. (2014) recently demonstrated that microglia remain dependent on CSF1R for survival in adult animals. Pharmacological inhibition of CSF1R yields complete ablation (>99%) of microglia within 21 days. This approach is practical because it requires no mouse breeding and microglial depletion can be maintained as long as the drug is administered. Depletion of the microglial pool disturbs CNS homeostasis, and while neurons do not regenerate, microglia have significant potential to self-renew through proliferation. The incomplete depletion (80%) accomplished in CX3CR1^CreER^Rosa26^DTR^ mice is quickly recuperated by hyper-proliferation of surviving microglia (Bruttger et al., 2015). Even more efficient depletion (99%) using the CSF1R-inhibitor is followed by such rapid repopulation that the existence of a microglia-progenitor has been suggested (Elmore et al., 2014). A recent report, however, demonstrates the relatively high turnover of microglia in the steady-state, arguing against the existence of a microglia progenitor (Tay et al., 2017). Conversely, using the CD11b-HSVTK model repopulation occurs from peripheral myeloid cells (Varvel et al., 2012; Prokop et al., 2015).

Given this historic perspective of microglia depletion research, we review below the lessons learned using these different strategies, focusing on models of neuroinflammation (experimental autoimmune encephalomyelitis, EAE), acute neurodegeneration (stroke, toxin-induced neurodegeneration) or chronic neurodegeneration (Amyotrophic Lateral Sclerosis (ALS), Alzheimer’s disease, Prion disease). We do not review the role of microglia in development or homeostasis and refer the reader to separate reviews addressing these areas of research (Greter and Merad, 2012; Nayak et al., 2014).

## Amyotrophic Lateral Sclerosis

ALS is a generally fatal neurodegenerative disease characterized by progressive paralysis of skeletal muscles associated with motor neuron death and glial activation in the lumbar spinal cord. The most commonly used mouse model of ALS is based on the over-expression of human SOD1 carrying the G93A mutation (mSOD1 mice). Mice accumulate mutant SOD1 aggregates in the spinal cord, leading to motor neuron death and recapitulation of the central aspects of ALS pathology including an age-dependent accumulation of CD11b^+^ (Alexianu et al., 2001; Gowing et al., 2008), CD68^+^ (Henkel et al., 2006; Beers et al., 2011), MHC II^+^ (Hall et al., 1998), CD11c^+^ (Beers et al., 2011) microglia/macrophages in the spinal cord of mSOD1 mice.

That mutant SOD1 expression in microglia/macrophages contribute to disease progression has been convincingly demonstrated in the slow progressing SOD1G37R line in which CD11b-Cre-mediated removal of SOD1 from myeloid cells slowed progression of disease and extended survival (Boillée et al., 2006). Similarly, repopulating the empty microglial niche in mSOD1/PU.1^−/−^ mice with wildtype bone marrow extends survival compared to mice transplanted with mSOD1 bone marrow (Beers et al., 2006; Lee et al., 2012). It should be noted that one study reported no increase in motorneuron loss following partial (40%) elimination of microglia/macrophages during the symptomatic disease stage in CD11b-TK/mSOD1 mice (Gowing et al., 2008; Table 1).

**Table 1:** Chronic neurodegeneration models.

Disease setting	Depletion model	Effect	References
Amyotrophic lateral sclerosis	SOD1^G93A^/PU.1^−/−^ repopulated with WT bone marrow	Repopulation of empty microglial niche with WT extends survival compared to mice transplanted with mSOD^G93A^ bone marrow	Beers et al. (2006)
Amyotrophic lateral sclerosis	Gangciclovir to the spinal cord via osmotic pump in CD11b-HSVTK:SOD1^G93A^ mice	40% microglia reduction did not affect motor neuron loss	Gowing et al. (2008)
Amyotrophic lateral sclerosis	Clodronate depletion i.c.v in SOD1^G93A^ mice with WT repopulation	Microglial depletion significantly slowed disease progression and prolonged survival of the ALS mice but did not affect disease onset.	Lee et al. (2012)
Amyotrophic lateral sclerosis	α-Ly6C antibody in SOD1^G93A^ mice from symptom onset	Depletion of Ly6C^hi^ monocytes improved rotarod performance and extended survival	Butovsky et al. (2012)
Alzheimer’s disease	Clodronate i.c.v in 4-month old TgCRND8 mice	Depletion of perivascular macrophages reduced cerebrovascular amyloid deposition.	Hawkes and McLaurin (2009)
Alzheimer’s disease	Oral gangciclovir in 5-month old CD11b-HSVTK/APPPS1 mice	Microglial depletion affected neither amyloid plaque formation and maintenance or amyloid-associated neuritic dystrophy.	Grathwohl et al. (2009)
Alzheimer’s disease	Chimera using cytotoxic drugs and transplantation of Nr4a1^−/−^ bone marrow to APPPS1 mice	Depletion of Ly6C^low^ monocytes increased plaque load in cortex and hippocampus.	Michaud et al. (2013)
Alzheimer’s disease	Gangciclovir i.c.v via osmotic pump in CD11b-HSVTK/APPPS1 or CD11b-HSVTK/APP23 mice	Microglial depletion and repopulation from bone marrow in 3-month old amyloid-depositing mice had no effect on amyloid pathology after 3 months	Varvel et al. (2015)
Alzheimer’s disease	Gangciclovir i.c.v via osmotic pump in CD11b-HSVTK/APPPS1 mice	Microglial depletion and repopulation with bone marrow myeloid cells results in no net effect on amyloid beta pathology after 1 month.	Prokop et al. (2015)
Alzheimer’s disease	Clodronate depletion i.c.v or PLX3397 CSF1R inhibition (chow) in AAV-GFP/tau mice	Microglial depletion suppressed the propagation of tau and reduced excitability in the dentate gyrus	Asai et al. (2015)
Alzheimer’s disease	PLX3397 CSF1R inhibition (chow) for 28 days	In 10-month old mice a 90% reduction in non-plaque associated microglia and 50% reduction in plaque-associated microglia resulted in prevention of neuronal loss and improved memory. No effect on amyloid beta load. In 1.5-month old mice, no effect on amyloid pathology.	Spangenberg et al. (2016)
Prion disease	GW2580 CSF1R inhibition orally in mice injected with scrapie (ME7)	Microglial inhibition slowed the progression of chronic neurodegeneration and prevented development of the hyperactive behavioral deficits	Gómez-Nicola et al. (2013)
Prion disease	CCR2^−/−^ mice injected with scrapie (ME7)	Monocyte loss does not affect neuropathology or disease course	Gómez-Nicola et al. (2014)
Prion disease	Ganciclovir i.c.v via osmotic pump in CD11b-HSVTK and IL-34^−/−^ mice injected with scrapie (RML6)	Microglial depletion accelerated prion disease and reduced survival	Zhu et al. (2016)
Experimental autoimmune encephalomyelitis	Clodronate i.v in guinea pig spinal cord homogenate immunized pre-onset in Lewis rats	Macrophage depletion led to reduced clinical symptoms.	Huitinga et al. (1990)
Experimental autoimmune encephalomyelitis	Clodronate i.v MBP T cell adoptive transfer pre-onset in Lewis rats	Macrophage depletion led to reduced clinical symptoms and CNS inflammation.	Huitinga et al. (1995)
Experimental autoimmune encephalomyelitis	Clodronate i.v MBP T cell adoptive transfer pre-onset in SJL/J mice	Macrophage depletion led to reduced clinical symptoms and CNS inflammation.	Tran et al. (1998)
Experimental autoimmune encephalomyelitis	Ganciclovir i.p in CD11b-HSVTK mice pre-induction for MOG_35–55_ peptide EAE	Conditional paralysis of microglia delayed disease onset and in repression of clinical EAE signs through	Heppner et al. (2005)
Experimental autoimmune encephalomyelitis	α-CCR2 antibody i.p in CX3CR1^GFP/+^ mice pre-induction for MOG_35–55_ EAE	Depletion of CCR2^+^Ly-6C^hi^ monocytes strongly reduced central nervous system autoimmunity	Mildner et al. (2009)
Experimental autoimmune encephalomyelitis	Clodronate i.v at onset in C57BL/6 mice for MOG_35–55_ EAE	Long-term depletion of monocytes prevents worsening of neurological deficits and long-term axonal loss	Moreno et al. (2016)
Experimental autoimmune encephalomyelitis	CD169-DTR mice pre-onset for MOG_35–55_ EAE	Depletion of CD169^+^ cells markedly reduced neuroinflammation and ameliorated disease symptoms in EAE-affected mice.	Bogie et al. (2017)

Whether microglia or monocytes make up the majority of the activated macrophage population in the spinal cord is a matter of controversy. One elegant study using parabiotically connected GFP^+^ and mSOD1 mice recorded no parenchymal GFP^+^Iba-1^+^ cells in the spinal cord at the late stage of disease when microgliosis is extensive (Ajami et al., 2007). Similarly, a more recent study did not detect any Ly6C^+^ cells within the CD11b^+^CD45^+^ “microglial” compartment (Chiu et al., 2013). However, Butovsky et al. (2012) reported that Ly6C^hi^ monocytes progressively accumulate in the spinal cord (but not brain) of mSOD1 mice, reaching as much as 30% of the CD11b^+^ compartment in late stage animals, and that their antibody-mediated depletion attenuated motor neuron death and delayed disease onset and mortality. Such monocyte-specific contribution to disease progression is very interesting, since most previous studies have used targeting systems that do not discriminate between microglia and monocytes. However, this study would benefit from confirmation in other laboratories since the presence of spinal cord monocyte-derived macrophages is contradicted by parabiosis experiments (Ajami et al., 2007).

## Alzheimer’S Disease

Alzheimer’s disease is the most prevalent form of neurodegeneration and is characterized by the presence of amyloid beta plaques and neurofibrillary tangles. The recognition of a glial reaction as a hallmark of Alzheimer’s disease neuropathology was, despite being described by Alois Alzheimer himself (Alzheimer et al., 1995) long disregarded as an epiphenomenon. However, the recent discoveries that several immune and microglia/myeloid-expressed genes (i.e., CD33, CR1, TREM2) are genetically linked to Alzheimer’s disease has established the role of microglia in disease progression (Lambert et al., 2009; Griciuc et al., 2013; Guerreiro et al., 2013; Jonsson et al., 2013).

### Amyloid Beta

The *in vivo* microglial response to amyloid beta has been well characterized in amyloid-depositing mouse models. Microglia rapidly migrate to newly formed plaques (Meyer-Luehmann et al., 2008) where they display an altered morphology (Frautschy et al., 1998; Stalder et al., 1999) and upregulate a vast array of surface molecules (Bornemann et al., 2001; Frank et al., 2008). Whether microglia actively internalize amyloid beta fibrils in naive APP mice has been a matter of debate (Stalder et al., 2001; Bolmont et al., 2008), but the increasing amyloid load observed with age indicates that microglia are ultimately unable to control the amyloid burden. Direct proof that microglia do not limit either amyloid plaque formation or growth was provided by Grathwohl et al. (2009) who demonstrated that complete ablation of microglia for 4 weeks in either young or aged APPPS1 mice resulted in no net effect on amyloid beta burden or plaque-associated neuritic pathology (Table 1). This finding was replicated in the 5XFAD disease model using CSF1R inhibition to deplete microglia, which resulted in no difference in amyloid pathology in either young or old 5XFAD mice. Interestingly, however, this model is characterized by development of substantial neuronal loss with age and microglial depletion both prevented this neuronal loss and improved contextual memory. Microglial depletion was also accompanied by an attenuation of disease-driven inflammatory gene expression (Spangenberg et al., 2016).

These studies are important because they explain the observation that amyloid deposits progressively increase in APP mice despite the association of microglia. This is supported by evidence that microglia in the vicinity of amyloid beta plaques progressively lose phagocytic capacity (Hickman et al., 2008; Krabbe et al., 2013). However, it is important to understand that microglia can be stimulated into removing existing amyloid plaques by a variety of means (Boissonneault et al., 2009; Leinenga and Götz, 2015; Iaccarino et al., 2016; Daria et al., 2017).

Whether monocytes take part in the response to amyloid deposits is a matter of long-standing debate. Previous studies claimed that monocytes/peripheral myeloid cells possessed superior ability to remove amyloid deposits compared to microglia (Malm et al., 2005; Stalder et al., 2005; Simard et al., 2006; Butovsky et al., 2007). However, these studies were all based on whole body irradiation chimeras and exposure of the brain to irradiation has subsequently been elucidated to condition the brain for monocyte infiltration (Mildner et al., 2007). More recent studies using brain-protected irradiation chimeras (Mildner et al., 2011) or chemotherapy-induced myeloablation (Lampron et al., 2012; Michaud et al., 2013) have demonstrated very little engraftment of monocytes during disease progression in amyloid-depositing mice. Furthermore, complete exchange of the microglial compartment with peripheral monocytic cells did not affect amyloid beta burden (Prokop et al., 2015; Varvel et al., 2015), which was final proof that monocytes do not confer better amyloid beta removal ability than do microglia.

The possibility remains that monocytes take part in the removal of amyloid deposits in the cerebrovasculature, so-called cerebral amyloid angiopathy. Using two-photon *in vivo* imaging patrolling Ly6C^low^ monocytes have been observed to actively crawl on amyloid beta-laden cerebral blood vessels (Michaud et al., 2013). Ly6C^low^ monocytes are dependent on the transcription factor Nr4a1 for their survival (Hanna et al., 2012) and specific elimination of Ly6C^low^ monocytes by transplanting Nr4a1^−/−^ bone marrow into chemotherapy-myeloablated APP/PS1 mice significantly increased the build up of amyloid deposits (Michaud et al., 2013).

Lack of Ly6C^low^ monocytes could also explain why CCR2^−/−^APP mice have increased levels of amyloid deposition in cerebral blood vessels (El Khoury et al., 2007; Mildner et al., 2011). While the authors attributed increased amyloid buildup to loss of CCR2 in the perivascular myeloid compartment, involvement of Ly6C^low^ monocytes cannot be excluded. In fact, perivascular macrophages are not lost in CCR2^−/−^ mice (Goldmann et al., 2016), in contrast to Ly6C^low^ monocytes which are directly derived from Ly6C^hi^ monocytes and therefore are also significantly reduced in CCR2^−/−^ mice (Yona et al., 2013). Another study has attempted to more specifically address the role of perivascular macrophages in buildup of vascular amyloid deposits through i.c.v injection of clodronate to deplete CD163^+^ perivascular macrophages while sparing parenchymal Iba-1^+^ microglia, this procedure resulting in a 5-fold increase in cerebral amyloid angiopathy load (Hawkes and McLaurin, 2009).

### Tau

Microtubule-associated protein tau (MAPT, tau) is a microtubule-stabilizing protein that during the course of AD becomes hyperphosphorylated, a process leading to dissociation from microtubules and aggregation into paired helical filaments and formation of neurofibrillary tangles (Wang and Mandelkow, 2016). Tau aggregates follow a predictable pattern of neuron-to-neuron spreading in the brain (Braak et al., 2011) and a recent study addressed the possible role of microglia in this process. Asai et al. (2015) developed a simple but elegant model of tau propagation in wildtype mice by injecting tau-expressing adeno-associated virus into the entorhinal cortex and observed how tau aggregates spread to the nearby hippocampus in only 4 weeks. Depletion of microglia using either clodronate liposomes or CSF1R inhibition dramatically halted the propagation of tau into the hippocampus. Similar results were obtained by inhibiting exosome synthesis, suggesting microglia could seed tauopathy (Asai et al., 2015).

There is also evidence that microgliosis can precede deposition of insoluble tau in transgenic mice (Yoshiyama et al., 2007; Maphis et al., 2015), indicating that microglia could drive spatiotemporal tau propagation through production of neuroinflammatory mediators. Loss of CX3CR1 amplifies tau pathology in hTau mice in an IL-1β-dependent manner (Bhaskar et al., 2010) and tau hyperphosphorylation could be induced in wildtype mice by intracerebral transplantation of CX3CR1^−/−^ hTau microglia (Maphis et al., 2015). This was the proof-of-concept that reactive microglia could drive tau pathology.

## Prion Disease

Prion disease is a transmissible neurodegenerative disorder affecting both animals and humans. The contribution of myeloid subsets to disease progression has been characterized using mouse models in which prion disease is produced by intracerebral inoculation of scrapie protein strains. While microglial proliferation and activation occurs irrespective of the inoculated prion strain (Cunningham et al., 2005) and correlates with onset of neurological deficits (Boche et al., 2006) there is little evidence of monocyte infiltration during disease progression. Preventing monocytes from entering the circulation using CCR2^−/−^ mice does not exacerbate neuropathology or disease course in prion-infected mice (Gómez-Nicola et al., 2014; Table 1). Whether microglial proliferation observed during prion disease is beneficial or deleterious is a matter of conflicting data. Using a CSF1R inhibitor Gómez-Nicola et al. (2013) limited microglial proliferation which prevented neurodegeneration, improved behavioral impairments and extended survival in prion mice. Conversely, Zhu et al. (2016) reported that microglial depletion (CD11b-HSVTK) or reduction (IL-34^−/−^) exacerbated prion disease and reduced survival, suggesting a neuroprotective role for microglia. Since the two studies have used different scrapie strains, different strategies to limit the microglial response and different timing of the microglial targeting, it is difficult to consolidate the data sets. However, it could indicate strain-specific microglial responses or suggest that microglia possess neuroprotective and neurotoxic reactions to prions at different time points during the disease course.

## Experimental Autoimmune Encephalomyelitis

The development of demyelinating lesions that is characteristic of multiple sclerosis (MS) and its mouse model EAE is initiated by autoreactive, myelin-specific CD4^+^ T cells. These are reactivated within the CNS (Goverman, 2009), secreting factors that open the blood-brain barrier and recruit a heterogeneous population of myeloid cells that drive demyelination. The primary importance of myeloid cells in pathogenesis is indicated by the strong genetic association of MHC II and MS (Sawcer et al., 2011). While infiltrating monocyte-derived macrophages drive disease pathogenesis (as detailed below), the contribution of resident microglia during the different disease phases is still not completely understood.

It has been demonstrated that EAE severity correlates with numbers of monocytes infiltrating the spinal cord and that prevention of this infiltration protects against EAE development (Fife et al., 2000; Mildner et al., 2009; Ajami et al., 2011). Importantly, the presence of the infiltrating cells in the CNS is transient, their numbers decreasing significantly during the remission phase of EAE (Hesske et al., 2010; Ajami et al., 2011), leaving the niche occupied by resident microglia. It is evident from our review (Table 1), that the majority of depletion studies in EAE have focused on preventing monocyte infiltration into the CNS from the periphery, with EAE pathology and symptoms being abrogated irrespective of the manner in which this is accomplished. Compared to infiltrating monocytes, microglia display low levels of MHCII and co-stimulatory molecules during EAE (Vainchtein et al., 2014) and their MHCII expression is dispensable for EAE induction (Greter et al., 2005). While preventing microglial expansion (referred to as microglial paralysis) has been demonstrated to substantially ameliorate the clinical signs of EAE and to strongly reduce CNS inflammation (Heppner et al., 2005), recent data indicate an alternative role of microglia compared to monocytes. Using electron-microscopy to study microglia and macrophage-specific interactions with axons, monocytes were shown to initiate demyelination at nodes of Ranvier, whereas microglia appeared to scavenge myelin debris (Yamasaki et al., 2014).

Given that EAE is primarily a disease state caused by infiltrating monocytes, the role of microglia in driving pathogenesis is not major. However, the role of microglia in restoring CNS during remission (healing) phases, or their lack of achieving this role during chronic disease states, warrants further investigation.

## Stroke

Stroke is caused by severe occlusion or stenosis of a cerebral artery and is the second leading cause of death and disability worldwide. Brain resident microglia become activated by various cytokines and damage-associated molecular patterns which are released by necrotic or apoptotic tissues (Shichita et al., 2012). An inflammatory response is triggered during the early stages of brain injury, leading to an influx of peripheral immune cells including macrophages and neutrophils (Tanaka et al., 2003; Gelderblom et al., 2009). Different studies have been performed to characterize the role of resident microglia and infiltrating peripheral macrophages in various mouse and rat models of stroke, but their roles are still under debate and vary depending on the experimental model employed (Table 2).

**Table 2:** Acute neurodegeneration models—stroke and related conditions.

Disease setting	Depletion model	Effect	References
Subarachnoid hemorrhage	Post-operative clodronate i.c.v in C57BL/6 mice injected with autologous blood	Reduced neuronal apoptosis d7 after surgery but not d15. Reduced vasospasm d7 and d15	Hanafy (2013)
Intra-cerebral hemorrhage	α-CCR2 antibody i.p in C57BL/6 mice and Ccr2^−/−^ BM chimeras pre- injection with autologous blood	Ly6C^hi^ monocyte depleted animals displayed a significantly less severe left hemiparesis	Hammond et al. (2014)
Aneurysmal subarachnoid hemorrhage by filament perforation	Ganciclovir i.c.v in CD11b-HSVTK mice post-ASH	Microglial depletion resulted in significantly reduce neuronal death	Schneider et al. (2015)
Intracerebral hemorrhage	PLX3397 oral gavage CSF1R inhibition by 21 days prior to injection of collagenase or autologous blood in C57BL/6 mice	Reduced leukocyte infiltration in the brain and improved blood–brain barrier integrity	Li et al. (2017)
Neonatal focal arterial stroke	Clodronate i.c.v in neonatal rats or CCR2^RFP+/−^CX3CR1^GFP+/−^ mice	Microglial depletion exacerbated injury and induced hemorrhages at 24 h	Fernández-López et al. (2016)
MCAO ischemic inflammation and brain injury	Ganciclovir i.p in CD11b-HSVTK mice pre-stroke	Selective ablation of proliferating microglial cells exacerbates ischemic injury in the brain.	Lalancette-Hébert et al. (2007)
MCAO ischemic inflammation and brain injury	Mac-1-saporin i.c.v in Wistar rats pre- or post-stroke	Microglial depletion did not affect the number of neuroblasts exiting the SVZ or their migration in the striatum	Heldmann et al. (2011)
MCAO ischemic inflammation and brain injury	Clodronate i.c.v in neonatal Sprague-Dawley rats pre-stroke	Lack of microglia increased brain levels of several cytokines and chemokines already elevated by ischemia–reperfusion, and increased the severity and volume of injury	Faustino et al. (2011)
Transient MCAO or photothrombosis	CD11b-DTR mice or CCR2^−/−^ chimeric mice	Early depletion of monocytes dramatically increases rate of hemorrhages in both stroke models and worse performance in rotarod	Gliem et al. (2012)
MCAO ischemic inflammation and brain injury	Human umbilical cord blood (HUCB) mononuclear cell transplantation i.v post-stroke in Sprague-Dawley rats	Monocyte depletion prevented HUCB cell treatment from reducing infarct size while monocyte enrichment was sufficient to reduce infarct size	Womble et al. (2014)
MCAO ischemic inflammation and brain injury	Clodronate i.p in ICR mice pre-challenge	Peripheral macrophage depletion reduced the myelin damage and microglia activation, enhanced microvessel density in the peri-infarct region, attenuated brain atrophy, and promoted neurological recovery	Ma et al. (2016)
MCAO ischemic inflammation and brain injury	PLX3397 (chow) CSF1R inhibition in C57BL/6 mice post-challenge	Microglial depletion exacerbated neurodeficits and brain infarction	Jin et al. (2017)
MCAO remote filament brain injury	PLX3397 (chow) CSF1R inhibition in Cx3Cr1^GFP/+^ mice	Microglia depletion leads to dysregulated neuronal calcium responses, calcium overload and increased neuronal death	Szalay et al. (2016)
MCAO ischemic inflammation and brain injury	Clodronate i.p or α-CCR2 antibody	Depletion of Ly6C^hi^ monocytes increased mortality in monocyte-depleted mice likely due to clodronate toxicity. Specific Ly6C^hi^ depletion did not influence mortality nor infarct volume or rotarod performance	Schmidt et al. (2017)

Using macrophage depletion via clodronate liposomes, Gliem et al. (2012) reported that the early influx of peripheral macrophages prevents hemorrhagic infarct transformation in a model of middle cerebral artery occlusion (MCAO), with mice treated at early time points having increased peri-lesional hemorrhage. Similar results were obtained using CD11b-DTR mice for macrophage depletion in the same study. In contrast, Ma et al. (2016) demonstrated a positive effect of peripheral macrophage depletion on the stroke lesion following clodronate treatment, with decreased myelin damage and microglial activation as well as decreased brain atrophy and increased neurological recovery following MCAO. Likewise, antibody blockade of CCR2-infiltrating macrophages reduced early motor deficits following intracerebral hemorrhage (Hammond et al., 2014), again indicating a negative role of myeloid cells in this model. Yet another recently published study contradicts all the aforementioned studies and concludes that targeting monocytes/macrophages (using clodronate, anti-CCR2 antibody or M1/M2 transfer) had no therapeutic value in acute ischemic stroke but only on mortality (Schmidt et al., 2017). The discrepancies in these studies, even though the same disease model was used, can be explained due to the different time points of monocyte depletion. Both studies that reported a detrimental effect of monocyte depletion (Hammond et al., 2014; Ma et al., 2016) depleted monocytes before inducing MCAO whereas in the first study (Gliem et al., 2012) monocyte depletion was performed after stroke induction, suggesting a differing role of monocytes during the course of disease.

Numerous stroke studies have been performed using selective depletion of microglia instead of peripheral monocytes. Microglia seem to have a supporting role in various models of stroke, as exemplified by studies of neonatal focal arterial stroke. The i.c.v injection of clodronate liposomes before induction of the lesion yielded specific microglia depletion without affecting the periphery, resulting in increased local inflammation and injury severity as well as reduced vessel coverage that triggers hemorrhages in the injured brain regions (Faustino et al., 2011; Fernández-López et al., 2016). Microglia are therefore thought to contribute to the endogenous protection mechanisms of the brain during early stages after injury in neonates. Similarly, depletion of proliferating microglia using adult CD11b-HSVTK mice resulted in exacerbation of the stroke lesion, increased neuronal death and pro-inflammatory cytokine levels (Lalancette-Hébert et al., 2007). Depletion of microglia using a CSF1R inhibitor prior and subsequent to MCAO also exacerbated brain infarction and neurological deficits by promoting leukocyte infiltration into the brain and increased inflammatory cytokine levels in the area of the lesion, supporting the beneficial role of microglia in MCAO (Jin et al., 2017). Interestingly, using the same depletion method in the intracerebral hemorrhage model, microglia were concluded to have the completely opposite role in that model of brain injury. Depletion of microglia thus led to reduced lesion size, brain edema and neurodeficits, a lack of microglia attenuated leukocyte infiltration, decreased inflammatory cytokine levels and preserved the integrity of the blood brain barrier (Li et al., 2017). Most studies to date have focused on the effect of microglia/monocyte depletion during the acute phase of brain injury. However, the roles of microglia/monocytes can differ at various time points and between injury models, underlining the many potential roles of inflammatory monocytes and microglia in the pathogenic and regenerative CNS (Hammond et al., 2014).

## Toxin-Induced Neurodegeneration

Microglia can have multiple roles during acute hippocampal neurodegeneration (Table 3), as demonstrated using a model of diphtheria toxin-inducible neuronal loss. Microglia numbers increase dramatically after lesion formation, without evidence of peripheral myeloid infiltration. Elimination of microglia during this procedure exacerbated neuronal loss (Rice et al., 2015). However, elimination subsequent to lesion formation improved cognitive recovery and reduced inflammatory signaling (Rice et al., 2015), demonstrating a deleterious response of microglia *after* as opposed to *during* lesion formation, again stressing how important accurate timing in microglia depletion studies is to obtain comparable results between studies. In a subsequent study, the elimination and repopulation of microglia after the procedure was reported to similarly reduce neuroinflammation, improve behavioral recovery and synaptic densities (Rice et al., 2017). The role of microglia in these toxin-induced models appears to be time-dependent, with an acute pathogenic role becoming a return-to-homeostasis (healing) role at later timepoints.

**Table 3:** Acute neurodegeneration models—toxin-induced neurodegeneration.

Disease setting	Depletion model	Effect	References
Diptheria Toxin-induced hippocampal lesion	PLX3397 CSF1R inhibition (drinking water) in CaM/Tet-DTA mice post-lesion or during lesion	Post-lesion microglial depletion improves behavior (elevated-plus maze and morris-water maze) and reverses lesion-induced increase in inflammatory signaling. Microglial depletion during lesion exacerbates neuronal loss in hippocampus	Rice et al. (2015)
Diptheria Toxin-induced hippocampal lesion	PLX5622 CSF1R inhibition (chow) in CaM/Tet-DTA mouse post-lesion	Microglial elimination and repopulation, largely resolves chronic neuroinflammatory responses and improved behavioral abilities	Rice et al. (2017)
LPS-induced striatal neurodegeneration	Clodronate depletion i.v in gerbils	Attenuated striatal macrophage infiltration reduced the severity of LPS-induced neurodegeneration	Zito et al. (2001)
Parkinson’s disease	Clodronate i.v in MPTP (i.p) model of PD in C57BL/6	Partial depletion of peripheral Ly6C^hi^ monocytes does not affect basal ganglia TH^+^ neuronal loss but protected against loss of TH^+^ neurons in the myenteric plexus (enteric nervous system)	Côté et al. (2015)
Kainic-acid induced epilepsy	Clodronate i.p. in C57BL/6	Depletion of F4/80^+^ cells in hippocampus reduces survival of dentate gyrus granule neurons.	Zattoni et al. (2011)
Pilocarpine-induced epilepsy	Pilocarpine-induced epileptic seizures in CCR2^−/−^ mice	CCR2^−/−^ and WT mice develop similar seizure severity but CCR2^−/−^ mice develop less hippocampal neurodegeneration	Varvel et al. (2016)

While partial depletion of circulating monocytes did not affect MPTP-induced neuronal loss in the basal ganglia (Côté et al., 2015; a model of Parkinson’s disease), LPS-induced striatal neurodegeneration in gerbils was attenuated by similar means (Zito et al., 2001). Kainic acid- or pilocarpine-induced eplieptic seizures in mice provoke microglial activation in the hippocampus and a delayed entry of monocytes into the parenchyma (Varvel et al., 2016). Varvel et al. (2016) elegantly showed that monocytopenic CCR2^−/−^ mice developed less hippocampal neurodegeneration, suggesting a detrimental role of monocytes. However, peripheral clodronate administration reduced accumulation of CNS-infiltrating F4/80^+^ macrophages and reduced survival of dentate gyrus granule cells (Zattoni et al., 2011) indicating a beneficial role of monocytes.

## Injury

Acute injury can be inflicted on the CNS in different ways, be it by crush or cut in the spinal cord, or traumatic brain injury (Table 4) as typified by traffic accidents and more recently by professional American sportsmen (Cherry et al., 2016). What is often characteristic of these injuries is a chronic state of neuroinflammation that can be experienced years after the initial injury, and this is associated with permanent microglial dysfunction. Despite this variation in the type of injury model and in the method of microglial depletion that have been reported there are few effects of reduced microglia numbers during the responses in these settings.

**Table 4:** Acute neurodegeneration models—injury.

Disease setting	Depletion model	Effect	References
Partial sciatic nerve ligation	Mac-1-saporin i.t in C57BL/6 mice pre-ligation	Acute depletion of spinal cord microglia had no effect on mechanical or thermal activity nor on allodynia following PSNL injury	Yao et al. (2016)
Neuropathic pain in spinal nerve transection	CX3CR1^CreER^R26^DTR^ mice pre-injury	Depletion of microglia delayed but did not reverse neuropathic hypersensitivity after peripheral nerve injury	Peng et al. (2016)
Repetitive concussive traumatic brain injury	Valganciclovir i.c.v in CD11b-HSVTK mice pre-TBI	Microglial depletion did not affect the rate of neuronal death	Bennett and Brody (2014)
Spinal cord injury	Clodronate i.p/i.v in LysM^EGFP^ mice pre-acute compression injury	Macrophage depletion did not affect the extent of the microglial-based inflammatory response in the lesion	Mawhinney et al. (2012)
Spinal cord injury	Clodronate i.p in LysM^tdTom^ > CX3CR1^GFP^ chimeric mice post-mid-thoracic (T8) contusive injury	Macrophage depletion resulted in changes in multiple cytokines that make the injury site less fibrotic and more conducive to axonal growth	Zhu et al. (2015)

Thus in a model of repetitive brain concussion microglia did not contribute to acute axon degeneration after multiple concussive injuries, although there is still a possibility of longer-term effects on axon functionality (Bennett and Brody, 2014). However, in a mouse model of spine transection, resident microglia and peripheral monocytes were concluded to act synergistically to initiate hypersensitivity and to promote the transition from acute to chronic pain following peripheral nerve injury (Peng et al., 2016).

It is possible that the severity of the extensive trauma in many of these models affects too large an area to make the microglial loss significantly synergistic, or alternatively that the ensuing chronic microglial dysfunction only gradually develops over time.

## Other *In Vivo* and *In Vitro* Models of Depletion

Given the proven propensity of pharmacological agents to efficiently deplete microglia and macrophages, it is not surprising that these have been applied in additional *in vivo* and *in vitro* settings (Table 5). It is apparent that clinical cognitive decline is a long-term condition induced by both cranial irradiation (Acharya et al., 2016) and peripheral surgery (Degos et al., 2013), and in both settings the depletion of myeloid cells increases cognitive ability. While in the former setting an effect on microglia would be expected, and has recently been reported to differ depending on the age of the mice (Han et al., 2016), the latter is intriguing as a peripheral skeletal injury is seemingly a long distance away from the CNS. The interplay between peripheral macrophages and CNS-resident microglia is thus probably more intricate and extensive than we currently understand. Organotypic CNS slice cultures provide an alternative to live mice for study of CNS homeostasis and disease, and microglia depletion agents can be applied to these.

**Table 5:** Other *in vivo* and *in vitro* models.

Disease setting	Depletion model	Effect	References
Cranial irradiation	PLX5622 CSF1R inhibition (chow) in C57BL/6 mice	Elimination of microglia ameliorates radiation-induced cognitive deficits (novel object recognition, object in place, fear conditioning) but has no effect in non-irradiated mice	Acharya et al. (2016)
Post-operative cognitive decline	Clodronate i.p in CCR2^RFP/+^ CX3CR1^GFP/+^ mice with stabilized tibial fracture	Depletion of macrophages prevents hippocampal neuroinflammation and memory dysfunction	Degos et al. (2013)
NMDA-induced excitotoxic lesion in organotypic hippocampal slice cultures	Clodronate depletion in Wistar rat tissues	Microglial depletion increases the number of degenerating neurons after excitotoxic lesions	Kallendrusch et al. (2013)
Mixed neuronal cultures	Deoxyglucose-induced death in cell culture	Microglia death via inhibition of glycolysis and ATP depletion, inducing microglial necrosis and their phagocytosis by other microglia	Vilalta and Brown (2014)
Organotypic spinal cord slice culture	Clodronate depletion in Sprague-Dawley rat tissues co-cultured with neural progenitor cells	Depletion of microglia decreased the apoptotic rate of NPCs, more NPCs differentiated into neurons, and glial differentiation was impaired	Liu et al. (2013)

Generally the findings suggest that tissues devoid of microglia have less ability to protect neurons.

## Points of Perspective

Given the vast expansion in the field of microglia research during recent years it will be important to build on previous interpretations in the light of new knowledge. For example, the absence of any effect on amyloid beta burden by microglia depletion in Alzheimer’s disease models could be explained by recent results suggesting that the key function of amyloid plaque-associated microglia is to form a barrier around it (to compact the amyloid fibrils into a dense plaque) rather than solely their previously perceived role in amyloid phagocytosis (Yuan et al., 2016).

It has proven difficult to permanently deplete microglia in a specific manner and there are caveats with all the depletion models employed. The nature of the cell death of depleted microglia within the CNS is also an issue that has not been addressed, and this might not only vary between depletion systems, but might also trigger downstream (e.g., epigenetic) programmes that we are currently unaware of but which have significant bearing on the interpretation of the studies.

The enormous propensity of the CNS to repopulate with myeloid cells, be they surviving microglia colonies that hyperproliferate, or CNS-adapted infiltrating monocytes that fill up the available niche, indicates that a myeloid-deficient CNS is a highly non-physiological condition. While the infiltrating monocytes co-occupy the CNS and begin to express microglia-specific proteins, it still remains to be proven if they develop *full* microglial functionality. Exactly how the CNS regulates the repopulation process and senses when the niche is replenished are important unanswered questions.

What is clear from our review is that there is some disparity between the results of different microglia depletion systems in the same disease model. Discerning the underlying molecular mechanisms that lead to these different outcomes will serve to further our knowledge of the pathogenesis that we wish to target therapeutically. In particular, the relative timing of microglial depletion in relation to the insult might explain some of the disparity, and deserves further consideration.

While the homeostatic functions of microglia are well understood, these cells always having activity in surveying the CNS, the molecular basis for these functions are less well characterized. TGFβ appears to be a key cytokine in preventing microglia and other infiltrating myeloid cells from pro-inflammatory (pathogenic) activation (Buttgereit et al., 2016; Parsa et al., 2016). The existence of sub-populations in different regions of the CNS has been demonstrated (Grabert et al., 2016), and so it is plausible that even homeostatic microglia function varies between different CNS microenvironments (Harris, 2014). A very recent study using a novel fate-mapping strategy has described such regional differences of self-organization of mature microglial subpopulations during both health and disease in the CNS (Tay et al., 2017). If and when subpopulation-specific microglial markers can be defined then it might even be possible to deplete individual subpopulations in the future. The more refined our understanding of these issues becomes, the more likely we will be able to design efficient therapeutic paradigms.

As it is clear that microglia are implicated as a part of all the neurodegenerative diseases described in this review article, modulation of microglial functionality (suppression of pro-inflammatory, pathogenic properties) or replacement with CNS-adapting macrophages are intriguing prospects for immunotherapy. We have previously reported that immunosuppressive macrophages can down-modulate pro-inflammatory macrophage and T cell activities in settings of experimental MS (Zhang et al., 2014), indicating that immunomodulation with favorable clinical outcome is possible. With the knowledge that monocytes can become CNS-adapted during repopulation of a microglia depleted CNS, further effort in manipulating this phenomenon to a therapeutic end is warranted.

## Author Contributions

All authors have had similar roles in defining the literature to be reviewed, preparing individual sections of the text and working up the final version of the manuscript.

## Conflict of Interest Statement

The authors declare that the research was conducted in the absence of any commercial or financial relationships that could be construed as a potential conflict of interest.
